# Effectiveness of the U-Niko intervention: Protocol for a cluster randomized controlled trial of a municipal-based tobacco and nicotine cessation intervention for adolescents and young adults

**DOI:** 10.1371/journal.pone.0323514

**Published:** 2025-10-16

**Authors:** Sofie Bergman Rasmussen, Christina Bjørk Petersen, Mette Rasmussen, Nina Kamstrup-Larsen, Charlotta Pisinger

**Affiliations:** 1 Center for Clinical Research and Prevention, Bispebjerg and Frederiksberg Hospital, Frederiksberg, Denmark; 2 National Institute of Public Health, University of Southern Denmark, Copenhagen, Denmark; Federal University of Paraiba, BRAZIL

## Abstract

**Introduction:**

The use of nicotine products among youth is increasing, while existing cessation services remain underutilized. Thus, the U-Niko intervention has been developed to provide an evidence-based, youth-oriented approach for effective nicotine cessation.

**Methods:**

This protocol describes a two-arm cluster randomized controlled trial of youth aged 16–25 in 55 municipalities in Denmark. Prior to the study a stratified randomization was carried out using the online randomization program Sealed Envelope, allocating 27 municipalities to the U-Niko intervention group and 28 municipalities to the control group (tobacco and nicotine cessation recruitment and counseling as usual). The primary outcomes will measure the effectiveness of all three focus areas in the intervention group compared to the control group: A) the municipal counselors’ self-efficacy in youth cessation counseling, B) the number of recruited youths for cessation counseling, and C) the self-reported 14-day point prevalence of abstinence of youth at six months follow-up. Secondary outcomes are the number of recruited youths in a municipality compared to the previous year, continuous abstinence, and validated 14-day point prevalence of abstinence at six-months follow-up.

**Discussion:**

By evaluating all three focus areas of the U-Niko intervention, this study aims to provide robust evidence for improving youth cessation interventions at local and national levels.

**Trial registration:**

The study was registered in ANZCTR (ACTRN12624001470583) on 18/12/2024. The Universal Trial Number is U1111-13-14-6117.

## Introduction

In recent years, youth nicotine use has risen significantly, with nearly 36% of Danish youth (15–29 years) using at least one tobacco or nicotine product [1]. While more than half of young people who smoke and more than 70% of those who use oral nicotine products wish to quit [1], many struggle with high levels of nicotine dependence, making unassisted quitting difficult [2].

The detrimental effects of smoking extend beyond physical health, adversely affecting mental health and contributing to increased school absence among youths [3–5]. Furthermore, early exposure to nicotine in any form may yield long-term negative consequences for brain development [6–8] and has been found to function as a “gateway drug” to other psychoactive substances, including cocaine and cannabis [9–11].

Although Denmark offers cost-free, high-quality smoking cessation services, young people underutilize these services and have lower quit rates than adults [12]. Group-based smoking cessation counseling is the most frequently used and is very effective [13]; however, municipal counselors report limited experiences with youth-oriented quit services [12] and call for evidence-based strategies to better support young users.

Despite this growing need, there is a lack of high-quality studies on youth tobacco and nicotine cessation interventions, and the existing literature is inconclusive [14]. However, interventions based on social cognitive theory [15] and group-based smoking cessation counseling [16] seem to be effective in promoting longer-term abstinence in young adults. Regarding the cessation of nicotine products (not cigarettes): Low-grade evidence suggests that multi-component interventions that include counseling and peer elements and a text-based cessation program based on behavioral support may be effective in the cessation of nicotine products in youth. Pharmacotherapy seems to be well tolerated by young people, but no significant effect has been shown [17,18].

Given the rising use of nicotine products among youth, their difficulties in quitting unassisted, and the underutilization of existing cessation services, there is a pressing need for effective, evidence-based interventions tailored to young users.

This paper describes the study protocol for a cluster randomized trial evaluating the effectiveness of the U-Niko intervention (‘Unge uden nikotin’ – Youth without nicotine), a tailored tobacco and nicotine cessation intervention for youth. Developed for Danish municipalities, which are key stakeholders responsible for cessation efforts, the intervention aims to provide evidence for improving youth tobacco and nicotine cessation strategies.

## Methods

### Study design

This study is a cluster randomized controlled trial (RCT) including 55 Danish municipalities and aimed at youth aged 16–25. The intervention period will run from January 1, 2025, to December 31, 2025. The municipalities were randomly allocated to either the U-Niko intervention group or the control group in the spring of 2024. The youth participants will receive either the U-Niko intervention or a control condition (municipal cessation activities as usual, described under the section *Intervention*) based on the municipality in which they live, go to school, work, or, in some other way, have their daily activities. “Cessation activities” are defined as counselling processes over several sessions, not brief advice.

The study is a field trial in which all intervention activities are performed by municipalities, not researchers. The intervention municipalities will not receive any economic or manpower support from the research group.

**Fig 1:**
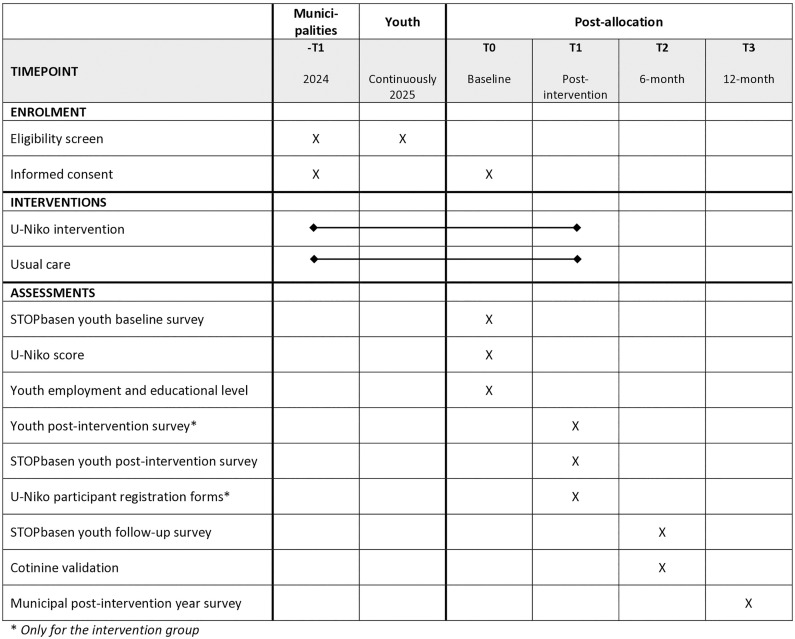
Time schedule of enrolment, interventions, and assessments on participant outcome inspired by the SPIRIT 2013 reporting guidelines.

Recruitment and cessation counseling will be conducted via the municipalities and will occur during the entire intervention year from January 1, 2025, to December 31, 2025. Enrollment and follow-up assessments will be conducted via the municipalities in cooperation with the national database on tobacco and nicotine cessation (STOPbasen) and the research group. STOPbasen is a Danish database where most cessation activities in Denmark are routinely registered and have high validity. Data collection is estimated to be complete in June 2026, with results expected to be ready to publish approximately 6–12 months later (Fig 1).

The study is conducted by the Center for Clinical Research and Prevention, Frederiksberg University Hospital, following the General Data Protection Regulation (GDPR) (EU) 2016/679, and was registered in ANZCTR on December 18^th^, 2024 (ACTRN12624001470583). The Universal Trial Number (UTN) is U1111-1314–6117. The Regional Health Research Ethics Committees in the Capital Region of Denmark waived the need for ethical approval of the project (no. F-24044815).

All youth receiving support to quit tobacco and/or nicotine products in the intervention and control municipalities will complete a short baseline survey at their first contact with the cessation activities, which will entail a written consent form for the study. The Danish Data Protection Authority and Danish Patient Safety Authority do not require parental permission for minors aged 15 years or older.

### Inclusion criteria

All Danish municipalities were invited to participate in the study. However, municipalities that participated in the development and feasibility study (n = 3) were excluded from the invitation to participate in the effect evaluation.

Eligible youth participants for the tobacco and nicotine cessation courses are youths aged 16 to 25 years who are currently (daily or occasionally) using at least one tobacco or nicotine product and wish for assistance to quit. Participants will be *excluded* from the study if they do not meet the listed inclusion criteria or fail to provide contact information and informed consent during enrolment and baseline assessment.

### Randomization

**Fig 2:**
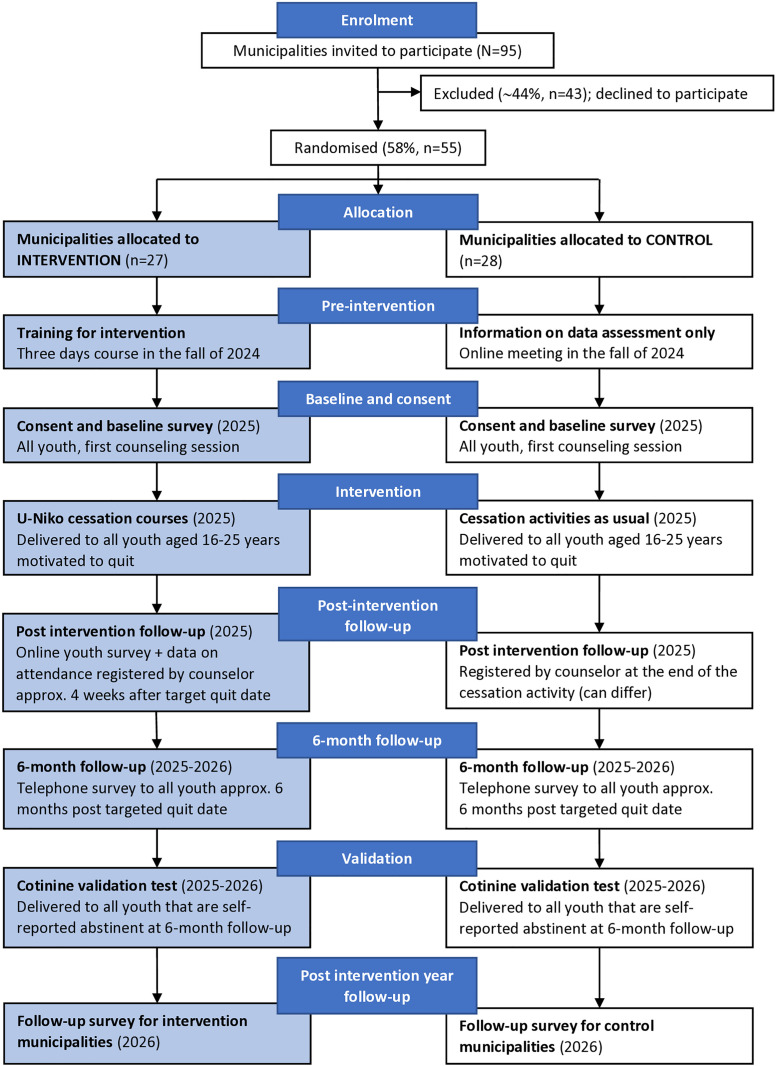
Overview of the intervention enrolment, preparation, trial activities, and data assessment.

All municipalities in Denmark that did not participate in the feasibility study (N = 95) were invited to register for the trial in March 2024. Of these, 55 municipalities (58%) accepted and were randomized into either an intervention or a control group using the online tool Sealed Envelope (Fig 2). Randomization was stratified by municipality size and inter-municipal cooperation (health clusters affiliation) to minimize the potential spill-over effect. Randomization was blinded to the researchers conducting the allocation. Participating municipalities differed from non-participants in several ways. Non-participating municipalities tended to be smaller (population < 50.000) and characterized by a higher average disposable income in 2024. In contrast, participating municipalities more frequently received funding from the Danish Health Authorities for tobacco and nicotine prevention and reported more cessation activities, including experiences with youths, in 2023. However, both groups had similar proportion of youth in the target group at the time of the invitation to participate. For a detailed comparison, see S1 Table in the Supporting Information.

### Sample size calculations

The sample size calculations are based on the expected difference in self-reported point prevalence abstinence (PPA) six months after the targeted quit between the intervention and control groups. Based on data from STOPbasen and international literature [14,19], the abstinence rate under the intention-to-treat analysis (ITT) approach is estimated to be 23% in the control municipalities and 34% in the intervention municipalities. The power calculations incorporate an intraclass correlation coefficient (ICC) of 0.05 to account for expected similarities among participants within clusters. This adjustment reflects the likelihood that young people within the same municipality receive support from the same youth coach/cessation counselor and are exposed to the same contextual environment (e.g., schools or other youth arenas). Despite extensive follow-up procedures, a 20% loss to follow-up is expected and is included in the sample size estimation [20]. Based on these assumptions, each municipality must include at least 27.8 youth participants in cessation activities during the entire intervention year, a total of 1,526 youth participants.

### Intervention

#### Theory of change.

The intervention is tailored to the unique needs of youth users of tobacco and nicotine products. It has been developed based on state-of-the-art research, insights from experienced Danish cessation counselors, and preferences of young tobacco and nicotine users in partnership with Rygestopkonsulenterne ApS (The smoking cessation consultants), a consulting firm with more than 20 years of experience with tobacco and nicotine cessation counseling (S2).

The intervention targets three focus areas:

A) Training of counselors who assist young smokers and nicotine product users to quit
B) Recruitment strategy for youth to tobacco and nicotine cessation services
C) Cessation course for youth who use tobacco and nicotine products.

**Fig 3:**
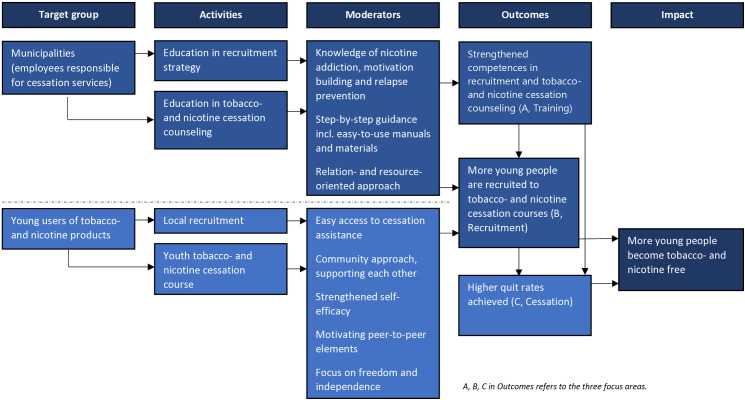
The program theory of change in the U-Niko intervention.

It is hypothesized that A) counselors trained through U-Niko will report higher skills and confidence in assisting youth quit attempts than those in control municipalities, B) intervention municipalities will recruit more youth into cessation services than controls, and C) youth participating in the intervention municipalities’ cessation services will have higher abstinence rates after six months than those in control municipalities (Fig 3). Furthermore, it is expected that there will be a synergistic effect by optimizing all three areas at the same time.

### The intervention municipalities

The municipalities will receive the U-Niko intervention, including online and physical materials, for all three focus areas. Table 1 provides an overview of the key elements of the intervention.

**Table 1:** Overview of the U-Niko intervention content.

Focus area	Key elements
A. Training	• Knowledge of nicotine addiction and relapse prevention • Knowledge and concrete tools to work with youth via behavioral and pedagogical approaches • Knowledge of motivation and ambivalence • Basic knowledge of novel tobacco and nicotine products and their harmful effects • Knowledge of the use of the U-Niko score and how to assess physical dependence • Instructing stress reduction exercises
B. Recruitment	• Step-by-step guidance and tools on how to contact and establish collaborations with youth arenas • Guidance and examples of how to promote the course on tobacco- and nicotine cessation in a motivating way using face-to-face interactions in the youth arena and tools such as videos and posters • Recommendations and examples on how to keep in touch with young people and strengthen their motivation from registration to attendance at the course • Tools and guidance on how to strengthen existing collaboration with a youth arena and how to expand to other arenas year by year
C. Cessation	• Group-based approach, strong focus on community • Commitment to complete cessation from all tobacco and nicotine products • Behavioral counseling • Strengthening of self-efficacy • Focus on a trust-based relationship, to make all participants feel safe • Peer elements and focus on sharing experiences • Understanding of own addiction and tools on how to overcome cravings • Stress reduction exercises • Text messages to S

A, B, C in refers to the three focus areas.

#### A. Training.

Training in the recruitment strategy is delivered through a 1-day course. During training, guidance is offered on how to engage with youth (face-to-face interaction with them in classes or at their workplace, sports clubs, campuses, etc.) and how to motivate them to participate in the course. Videos and posters featuring young peers (role models) sharing their reflections on smoking/nicotine use and desire to quit are provided.

Youth counselor training is delivered through a 2-day course. The youth counselor training program is aimed at both experienced smoking cessation counselors and individuals with a pedagogical background who have never worked with smoking cessation. It focuses on relational and resource-oriented behavioral techniques, knowledge of nicotine addiction, as well as on how to motivate youth to quit tobacco and nicotine and how to prevent relapse. The program focuses on a non-judgmental and supportive approach. Additionally, the training provides a foundational understanding of novel tobacco and nicotine products and their harmful effects.

#### B. Recruitment.

The recruitment strategy is developed as a step-by-step guidance on where to recruit and how to contact youth arenas for recruitment (i.e., schools, workplaces, fitness centers, sports clubs, campuses, barracks, and meeting places for youth), as well as how to encourage them to cooperate. The intervention group will receive free recruitment materials (videos and letters) and guidance on how to recruit in different youth arenas over time.

#### C. Cessation.

The cessation course consists of seven meetings planned over eight weeks. The sessions last between 1.5 hours and 45 minutes. It is advised that approximately twelve young people are recruited for each course established. Participating municipalities can recruit and create as many courses as they wish during the intervention year.

The course is anchored around key constructs from social cognitive theory and posits that learning occurs in a social context with a dynamic and reciprocal interaction of the person, environment, and behavior [21]. Peer elements are used both in recruitment and in group counseling. The approach to young people stems from theories of cognitive behavioral therapy [22], as well as narrative engagement theory [23]. Furthermore, the course is based on the International Child Development Programme (ICDP) and its eight interaction themes as a tool for relationship- and resource-oriented practices [24]. The exercises stem from mindfulness-based cognitive therapy [25] and mindfulness-based stress reduction [26].

As part of the cessation course, a newly developed score (The U-Niko score, validated by 25 young people, S3) is used to measure physical nicotine dependence. The score has been developed because the nationally used Fagerström score is not suitable for youth, since many young people use more than one tobacco and nicotine product, the consumption of e-cigarettes is difficult to measure, and the nicotine content in e-cigarettes and nicotine pouches can vary significantly. The U-Niko score consists of five short questions ranging from to 0–9, with 9 being the highest. A score of ≥ 6 indicates high physical nicotine dependence, suggesting that nicotine patches may be beneficial as a part of the cessation process. For individuals aged 18 + years with a nicotine patch allergy, cytisine is recommended for those with a score of ≥6.

### The control municipalities

The municipalities in the control group will continue their recruitment practices and their youth cessation activities “as usual.” The control municipalities can offer individual or group-based counseling of any duration.

The municipalities are not provided with any information about the intervention or any assistance to improve their recruitment strategies, training of counselors, or youth-oriented cessation activities during the intervention year. However, the control municipalities will be instructed on how to register baseline and follow-up information and will be offered information on how to use the U-Niko score, as the goal is to compare the young participants’ nicotine dependence uniformly in the intervention and control groups. Information on how to use the U-Niko score was delivered via an online meeting in December 2024 (Fig 2).

### Measures and data assessment

The data assessment is ongoing through 2025 and approx. six months into 2026, includes data on the effectiveness of all three focus areas of the U-Niko intervention and involves seven different data sources (Fig 1). Each focus area of the intervention has its own primary outcome.

#### A. Training.

In January 2026, the year after the intervention year, all municipal counselors from both intervention and control municipalities will receive an online survey examining their self-perceived competencies in working with youth tobacco and nicotine cessation using a version of the Short Occupational Self-Efficacy Scale (Short-OSE Scale) adapted to the cessation counseling context and translated into Danish (a scale from to 0–30, 30 being the highest level of perceived self-efficacy) [27].

The primary outcome is *the effect of the training of new youth counselors*, assessed through differences in counselor self-efficacy between the intervention and control municipalities.

#### B. Recruitment.

Data on the number of recruited youths will be registered in STOPbasen. The primary outcome is t*he effect of the recruitment strategy,* which will be assessed by comparing the number of recruited youth participants in the intervention municipalities with the control municipalities.

The secondary outcome is the *change in the number of youth recruited* for cessation activities in the intervention group between the intervention year 2025 and the previous year 2024

#### C. Cessation.

At the first cessation session, all participants in both the intervention and control municipalities will complete a baseline questionnaire on contact information, personal characteristics, information on tobacco and/or nicotine use, and the U-Niko score. The baseline questionnaire also contains a consent form for participation in the study. The baseline questionnaire will be registered in STOPbasen.

Further, the young participants in the intervention group will be invited to answer a short online survey on their experiences with the intervention after the course has ended (4 weeks post-target quit date), regardless of their tobacco and nicotine status.

Six months after the targeted quit date, all participants (in both the intervention and control groups) will be contacted by phone to examine the long-term effects of the cessation offer. A research assistant will contact the participants multiple times by phone and send text messages to decrease the number of missing values at follow-up.

All participants will be informed that each completed questionnaire (baseline survey, post-intervention survey (only intervention group), and six-month follow-up survey) will be entered into a raffle with the chance to win a gift certificate worth 1000 DKK (~134 euros/139 USD). In total, six participants from the intervention municipalities and four from the control municipalities will be drawn to win.

Participants who report being tobacco- and nicotine-free at the six-month follow-up will be invited to undergo a saliva cotinine test to validate their abstinence. The saliva test will be mailed to each participant who has agreed to participate and will be conducted via an online interview using Teams. The participant will take the test and show the answer to the research staff during the online meeting, discard the test, and receive a gift certificate for 300 DKK (~40 euros/42 USD), regardless of the outcome of the validation test.

The primary outcome is *the effect of the cessation course,* which will be measured using *long-term self-reported PPA* (not using any tobacco or nicotine product in the last 14 days) six months after the targeted quit date in the intervention municipalities compared with the control municipalities. To assess this, participants will be asked, “Have you used any tobacco or nicotine products in the last 14 days?” (Yes/No).

The secondary outcomes are *short-term self-reported abstinence* (i.e., at the end of the cessation course/counseling), *continuous self-reported abstinence* (from targeted quit date to six-month follow-up), and v*alidated PPA,* which will be assessed in all participants who report being tobacco and nicotine abstinent at the six-month follow-up and agree to participate in the cotinine validation process.

### Additional measures

Additional measures of the participants will be assessed in the baseline survey, including demographic information such as age, sex, gender, socioeconomic status, U-Niko dependence score, and product use (type, frequency, and duration). Furthermore, data on the employment of youth participants at baseline will be obtained from the Danish Education Register.

Additional measures for youth cessation counselors will be assessed during the post-intervention municipal follow-up survey. These measures include education and previous experience with tobacco and nicotine cessation activities.

### Data analysis plan

#### A. Training.

A table will present the characteristics of the counselors separately for the intervention and control municipalities, including gender, age, education, years of experience with youth, and experience with tobacco and/or nicotine cessation. The effect of training new youth counselors will be assessed by comparing their self-efficacy scores in the intervention and control municipalities after the intervention year. Because counselors are nested within municipalities, and municipalities are the unit of randomization, a linear mixed-effects model (LMM) with a random intercept for municipalities will be used to account for clustering. The model will be adjusted for counselor characteristics, such as counselor education, years of experience working with youth, and years of experience in tobacco and/or nicotine cessation.

Results from the LMM will be reported as estimated mean differences with 95% confidence intervals (CIs), and a p-value < 0.05 will be considered statistically significant.

#### B. Recruitment.

A table will present and compare relevant covariates between the intervention and control municipalities, including municipality size, socioeconomic indicators, funding received from the Danish Health Authority, previous experience with youth cessation counseling, and youth population. The effect of the recruitment strategy will be assessed by comparing the number of youths recruited for cessation activities in the intervention and control municipalities using an LMM. The model will be adjusted for relevant covariates, including the number of youths recruited in each municipality in the previous year (2024), municipality size, and whether the municipality received funding from the Danish Health Authority for youth-related cessation activities.

Results from the LMM will be reported as estimated mean differences with 95% CIs, and a p-value < 0.05 will be considered statistically significant.

Secondary results describing the absolute change in the number of youths recruited for cessation activities between 2024 and 2025 in intervention municipalities will be presented using descriptive statistics in a table, with the results shown separately for each municipality.

#### C. Cessation.

The analyses of the primary outcome on self-reported 14-days PPA at six months follow-up will follow the intention-to-treat (ITT) principle, assuming that all non-respondents are currently smoking or using nicotine. Data will be analyzed using a generalized linear mixed model (GLMM) to account for clustering, recognizing that youth participants within the same municipality may be more similar to each other than to participants from other municipalities. Results will be presented in a table as odds ratios (ORs) for 14-day PPA at six months after the targeted quit date, with corresponding 95% CIs. A p-value < 0.05 will be considered statistically significant.

Secondary outcomes, including short-term self-reported abstinence, continuous self-reported abstinence, and validated 14-days PPA at six months follow-up, will be analyzed using the same GLMM approach as for the primary outcome and presented in tables as ORs with corresponding 95% CIs. In addition, a table will present and compare the baseline characteristics of all youth participants between the intervention and control groups, including age, educational level, type of nicotine product used, and follow-up participation rates.

Given the high degree of data completeness in STOPbasenwe intend to employ a complete case analysis, excluding patients with any missing values in the included variables from the analysis (listwise deletion). Consequently, a sensitivity analysis using an as-observed approach will be conducted, including only participants with a valid follow-up. Sensitivity analyses will be performed using the final adjusted model from the main analyses. This approach builds on the assumption that data is Missing Completely At Random (MCAR). However, when it comes to smoking status, data are more likely to be Missing Not At Random (MNAR). Therefore, if necessary, considering the number of missing values and loss to follow-up, a sensitivity analysis using a chained multiple imputation model will be conducted based on the final adjusted model in the main analyses.

## Discussion

This paper describes the study design for evaluating the effectiveness of the U-Niko intervention, a national municipal-based tobacco and nicotine cessation intervention for Danish youth aged 16–25. By integrating insights from field experts, the experiences and preferences of young tobacco and nicotine users, and state-of-the-art research, this intervention aims to serve as an effective supplement to the existing cessation services in Denmark.

The U-Niko intervention is a complex intervention that encompasses three intervention focus areas: training, recruitment, and cessation. Evaluating each focus area independently allows for a nuanced assessment of the intervention’s effectiveness across its focus areas, and ensures that the contribution of each area is captured [28]. If some or all focus areas are shown to be effective, we plan to perform an impact evaluation including cost-effectiveness for the overall intervention and/or effective focus areas. This approach will enable policymakers and practitioners to identify which components are most effective, informing potential scaling and implementation strategies.

The study is a nationwide field trial, extending across the country, counting 55 municipalities from both rural and urban areas of Denmark. This is a great strength, since the intervention will be tested among youths with very different socioeconomic and cultural backgrounds.

Another strength is that it is a field trial and is not driven by researchers. If the intervention activities prove to be effective and municipalities find them helpful, meaningful, and easy to use, they will continue to use them. Thus, there is a high probability of sustainability after the end of the intervention year. By testing the effectiveness of the intervention in 27 different municipal settings, we can acknowledge context-dependent variables, such as social, organizational, political, and economic features, that influence intervention effectiveness [28]. As more than half of the municipalities in Denmark test the U-Niko intervention, it has received great attention, both from not-included municipalities and from Health Authorities.

Because the study is a field trial and the intervention occurs in a real-life setting, there are also some uncertainties regarding the comparison groups, data registration, and follow-up [29]. The youth-oriented cessation activities in the municipalities have been very sparse in the last few years, and there is a risk that only a few young persons will be included in 2025, resulting in too low a power to detect significant differences between the intervention and control municipalities. However, in early 2024, the Danish Health Authorities launched a call in which municipalities interested in improving their work with youth tobacco and nicotine cessation activities could apply for funding. In total, 58 million DKK (~7,7 million euros) were allocated to this work and funding must be used between fall 2024 and 2027. This may have a negative impact on the study, as some municipalities will have a much higher activity level than anticipated. Nonetheless, both the intervention (~44%, n = 12/27) and control municipalities (~54%, n = 15/28) received funding, and therefore, the hopefully positive effect of the increased resources will be present in both the intervention and control groups. Therefore, it should be feasible that the calculated sample size requirements on 27.8 participants during the whole intervention year in each municipality can be accommodated. The analysis of the effectiveness of the U-Niko intervention (recruitment and cessation) will consider that some of the municipalities (more control municipalities) have received extra funding and, therefore, have more resources.

Furthermore, the comparison between municipalities that agreed to participate and those that did not highlights important contextual factors that should be kept in mind when interpreting study findings and considering the external validity of the trial. While both participating and non-participating municipalities had on average the same proportion of youths in the target population, indicating a similar level of need, the municipalities that chose to participate were generally larger, and reported more prior experience with cessation activities. Furthermore, municipalities that participated in the study received higher funding from the Danish Health Authority for youth tobacco and nicotine prevention. However, this funding was announced only after the municipalities had enrolled in the study, and they were informed of their funding status only after randomization had been completed. Nonetheless, these differences suggest that participating municipalities may have had greater capacity to implement and deliver cessation interventions. This raises a concern regarding scalability. Although non-participating municipalities have a comparable youth population and thus similar needs, their more limited resources and experience may pose challenges for implementing the intervention in these settings. This should be considered when interpreting the trial’s potential for broader application.

Another uncertainty is that the municipalities have the responsibility to ensure that each young participant completes the baseline survey and consent form, which can be forgotten, and follow-up surveys may not be prioritized by the young participants. Further, data rely mostly on self-reported measures, which may be subject to reporting biases. However, to motivate higher participation rates in follow-up activities, all youth participants will enter a competition to win a gift certificate for 1000 DKK (~134 euros/139 USD) each time they finish a survey. In total, ten youth (six from the intervention group and four from the control group) will win a gift card. Financial incentives to improve youth survey participation rates are known to be very effective [30], but should be used in a way that will not influence young people’s answers (i.e., only be able to receive the gift certificate if you are tobacco and nicotine-free). Since participating in the competition to win a gift certificate is not dependent on whether the young person is still using tobacco or nicotine, it is anticipated that it will not result in any biases. Furthermore, all youth who state to be tobacco and nicotine abstinent and agree to participate in the cotinine validation after the six-month follow-up will receive a gift certificate for 300 DKK (~40 euros/42 USD) regardless of the test outcome. By offering these incentives to participate in the surveys, it is expected that validity will increase, and fewer young participants will be lost to follow-up.

The U-Niko intervention is resource-intensive, especially due to the face-to-face counseling. Previous studies have shown that low-intensity interventions may be effective. For example, a text message-based cessation intervention targeting youth users of e-cigarettes was shown to be both effective and cost-effective [31]. However, a more recent study suggests that face-to-face counselling yields better cessation outcomes compared to this text-based program, indicating that while digital interventions can be beneficial, in-person support may offer additional advantages in terms of effectiveness [32]. In the development stages of the intervention (S2), both youths who had received cessation counseling and those who had never tried any form of cessation counseling pointed out that face-to-face counseling would have a greater impact on their motivation to quit than online or automated text-generated counseling. With an increasing number of young people using tobacco and nicotine products, there is also a greater demand for effective cessation counseling [1]. Young people have more unassisted quit attempts than adult smokers but have lower success rates [33]. Providing access to cessation support is a key measure in the WHO Framework Convention on Tobacco Control (FCTC) [34]. Therefore, youths who are unable to quit on their own should be offered the help they need and deserve.

## Supporting information

S1 Comparison of participating and non-participating municipalities. (DOCX)

S2 Development of the U-Niko Intervention. (DOCX)

S3 Development of the U-Niko score. (DOCX)

S4 SPIRIT Fillable-checklist 15 Aug 2013. (PDF)

S5 U-Niko protocol application 2024. (PDF)
