# Supplementary material for: Effectiveness of the U-Niko intervention: Protocol for a cluster randomized controlled trial of a municipal-based tobacco and nicotine cessation intervention for adolescents and young adults
Source: PLoS One. 2025 Oct 16;20(10):e0323514. doi: 10.1371/journal.pone.0323514 (PMC12530545; doi:10.1371/journal.pone.0323514)
Supplement: S1 — (DOCX) [file pone.0323514.s001.docx]

# S1: Comparison of participating and non-participating municipalities

**Table S1: Demographic Variables between participating and non-participating municipalities**

|  | Participating  municipalities | Non-Participating municipalities |
| --- | --- | --- |
| No. of municipalities | 55 (57.9%) | 40 (42.1%) |
| Population size 1/1/24 (average) ^a^ | 73792 | 42973 |
| *Big, >70000 (%)* | 18 (33 %) | 2 (5 %) |
| *Medium, 50000-70000 (%)* | 14 (25 %) | 5 (12 %) |
| *Small, <50000, (%)* | 23 (42 %) | 34 (83 %) |
| Average yearly disposable income per person in 2024 ^b^ | 568,900.26 | 402,911.22 |
| Danish Health Authorities Funding ^c^ | 49.1% | 25% |
| *Amount received from the Danish Health Authorities** | 1,957,631.5 | 519,062 |
| Cessation activities in 2023 ^d^ | 94.5% | 85% |
| Self-reported experience with youth cessation acitivities ^e^ |  |  |
| *Large / very large experience* | 23.6% | 11.8% |
| *Little / very little experience* | 68.9% | 63.2% |
| *No experience* | 7.5% | 25% |
| Proportion of youth (16-25 years) ^f^ | 12.4% | 12.4% |

*^a^ Statistics Denmark – StatBank.dk/FOLK1A [28/8/25]
^b^ Statistics Denmark – StatBank.dk/INDKP106 [28/8/25]
^c^ Sundhedsstyrelsen - [www.sst.dk/da/puljer/Pulje-til-udvikling-af-ryge--og-nikotinstoptilbud-til-boern-og-unge-2024-2027](http://www.sst.dk/da/puljer/Pulje-til-udvikling-af-ryge--og-nikotinstoptilbud-til-boern-og-unge-2024-2027) [28/8/25]
* Per receiver, in DKK
^d^ STOPbasen yearly report 2023-2024 - <https://www.stopbasen.dk/_files/ugd/e0f865_0c0b4614ea4b4bafab2268d2a5eec897.pdf> [28/8/25]
^e^ Rasmussen SKB, Pisinger C. Nationwide experiences with youth-targeted smoking and nicotine product cessation. Tob Prev Cessat. 2023;9(August):1-13. doi:10.18332/tpc/169498*
*^f^ Statistics Denmark – StatBank.dk/FOLK1A [28/8/25]*
