# Supplementary material for: Effectiveness of the U-Niko intervention: Protocol for a cluster randomized controlled trial of a municipal-based tobacco and nicotine cessation intervention for adolescents and young adults
Source: PLoS One. 2025 Oct 16;20(10):e0323514. doi: 10.1371/journal.pone.0323514 (PMC12530545; doi:10.1371/journal.pone.0323514)
Supplement: S2 — (DOCX) [file pone.0323514.s002.docx]

# **S1: Development of the U-Niko Intervention**

Initially, a national survey was conducted to assess the experiences and practices of Danish municipalities concerning tobacco and nicotine cessation among young individuals [1]. This was followed by a workshop involving counselors from all municipalities who reported having substantial experience in youth counseling (N=14), as well as individual interviews with selected experienced counselors (N=4). Additionally, observations were made of several smoking and nicotine cessation courses for young people (N=14 meetings, approximately 18 hours), and interviews were conducted with several young individuals (N=7) to identify key components for motivating and maintaining abstinence from the youth's perspective. Furthermore, state-of-the-art international research on tobacco cessation in adolescents and young adults was compiled, and a contribution was made to a systematic review of nicotine product cessation in this demographic [2]. Based on this comprehensive knowledge, a prototype for the U-Niko intervention was developed.

**The foundation for the U-Niko prototype and intervention**


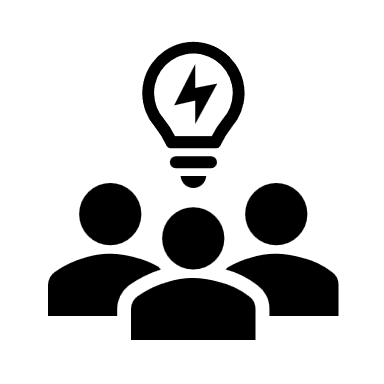

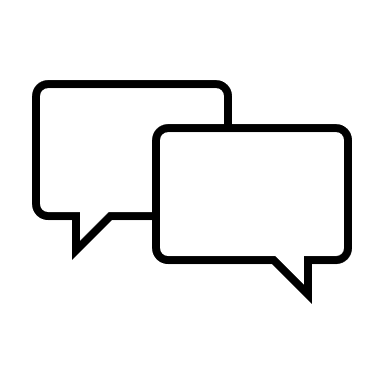

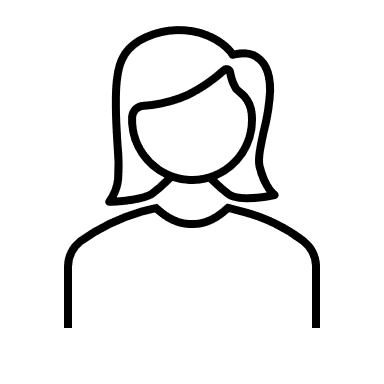

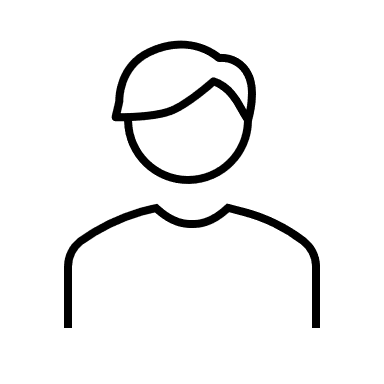

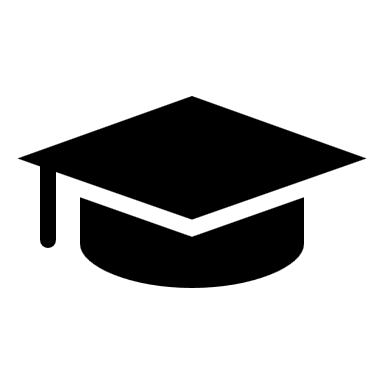

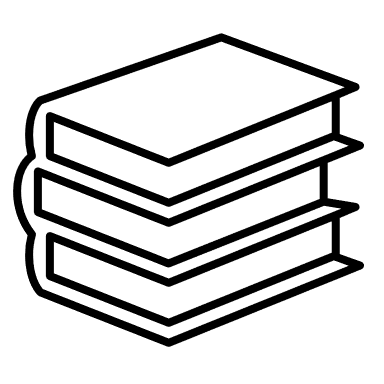


National survey

Workshop with counselors

Interviews with counselors, adolescents, and young adults

Systematic review


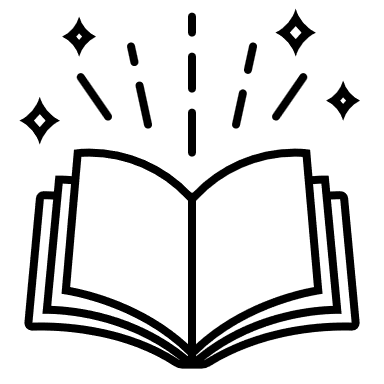

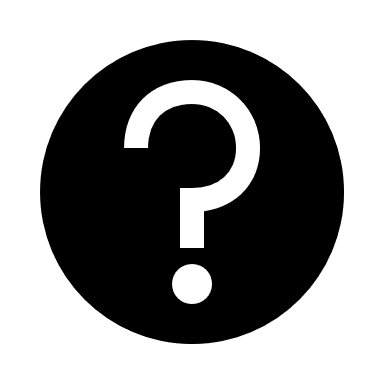

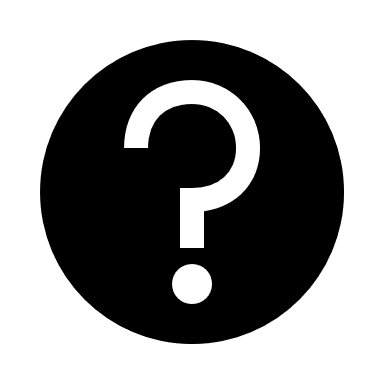

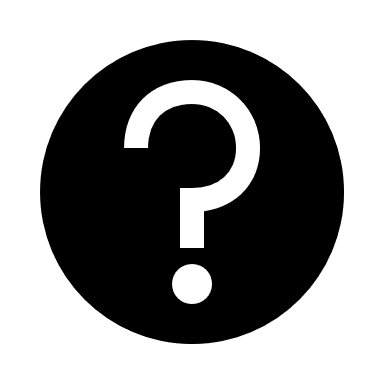


The prototype was evaluated across three municipalities from November 2023 to May 2024 to ascertain the acceptability and feasibility of its activities within a Danish municipal context. The feasibility study encompassed observations of the U-Niko youth cessation course (N=16 meetings, approximately 20 hours) and interviews with municipal youth counselors (N=3) as well as some youth participants (N=8). Based on feedback from the test municipalities and the young participants involved in the feasibility study, the interventions were refined to better address the needs of both counselors and youth. Subsequently, the intervention program underwent an expert review to ensure alignment with the latest scientific evidence and the Danish Health Authorities' requirements for best practices in tobacco and nicotine cessation counseling [3, 4].

**References**

[1] Rasmussen SKB, Pisinger C. Nationwide experiences with youth-targeted smoking andnicotine product cessation. *Tob Prev Cessat* 2023; 9: 1–13.

[2] Rasmussen SB, Pisinger C. Nicotine product cessation interventions in young adults: a systematic review (Revised version submitted: 03/06/2024). *Tob Use Insights*.

[3] *Behandling af nikotinafhængighed: Krav, anbefalinger og metoder til stoprådgivere*. Sundhedsstyrelsen, 2024.

[4] *Forebyggelsestilbud til borgere med kronisk sygdom: Kvalitetsstandarder*. Sundhedsstyrelsen, 2024.
