# Supplementary material for: Effectiveness of the U-Niko intervention: Protocol for a cluster randomized controlled trial of a municipal-based tobacco and nicotine cessation intervention for adolescents and young adults
Source: PLoS One. 2025 Oct 16;20(10):e0323514. doi: 10.1371/journal.pone.0323514 (PMC12530545; doi:10.1371/journal.pone.0323514)
Supplement: S3 — (DOCX) [file pone.0323514.s003.docx]

# S2: Development of the U-Niko score

Since the emergence of novel tobacco and nicotine products, the Fagerström score for Nicotine Dependence is no longer suitable for individuals who use more than one tobacco or nicotine product. This is because the consumption of e-cigarettes is difficult to measure, and the nicotine content in e-cigarettes and nicotine pouches can vary significantly. Therefore, there is a need for a new score that accounts for new products and consumption patterns and is developed to uncover dependence on future novel tobacco and nicotine products.

The development of the U-Niko score began in 2023 with a review of state-of-the-art research on measuring youth nicotine dependence through a questionnaire [1, 2] and followed the initial steps in developing and validating questionnaires in research [3, 4]. Afterward, an expert panel consisting of a professor in Tobacco and Nicotine Prevention and two cessation consultants with many years of experience brainstormed possible questions, eventually deciding on six preliminary questions (Table S1).

**Table S1: Preliminary questions of the U-Niko score (in Danish)**

| 1. Ryger du, eller bruger du andre nikotinprodukter dagligt?  *Do you smoke or use other nicotine products daily?* | - Yes - No |
| --- | --- |
| 1. Hvor lang tid går der, før du ryger, eller bruger andre nikotinprodukter, når du vågner om morgenen? *How soon after waking up do you smoke or use other nicotine products?* | - 0 – 15 min. - 15 min. 1- hour - More than 1 hour |
| 1. Ryger du, eller bruger du andre nikotinprodukter, lige inden eller efter du er gået i seng om aften?  *Do you smoke or use other nicotine products right before going to bed or after getting into bed at night?* | - Yes - No |
| 1. Ryger du, eller bruger du andre nikotinprodukter, steder hvor du ikke må?  *Do you smoke or use nicotine products in places where it’s not allowed?* | - Yes - No |
| 1. Føler du, at du har svært ved at koncentrere dig, bliver rastløs, nedtrykt og/eller irritabel, når du ikke har røget eller fået nikotin i et stykke tid? *Do you feel it’s hard to ocncentrate, or du you become restless, down, and/or irritable when you haven’t smoked or had nicotine for a while?* | - Yes, always - Yes, sometimes - No |
| 1. Føler du selv, at du er afhængig af nikotin?  *Do you feel that you are addicted to nicotine?* | - Yes - No |

The questionnaire where then pilot-tested in the fall of 2023 through mini-interviews with 20 adolescents and young adults aged between 16 and 23 years old in Copenhagen, and questions that showed to not be relevant or difficult to understand by the target group were reformulated or discarded.

This resulted in 5 questions (Table S2) which were tested in early spring of 2024 during the U-Niko feasibility study, where three municipalities used the score during their cessation counseling. In total 27 adolescents and young adults participated in the feasibility municipalities cessation counseling and filled out the U-Niko score. The counselors were instructed to offer nicotine plaster to those with a score ≥ 6. The feasibility evaluation showed that for the counselors who stuck to the instructions, the U-Niko score had been very helpful in tracking the youths who had a high physical dependence on nicotine and would experience withdrawal in the cessation process.

**Table S2. Final questions of the U-Niko score (in Danish)**

| 1. Ryger du, eller bruger du andre nikotinprodukter dagligt? *Do you smoke or use other nicotine products daily* | - Yes - No |
| --- | --- |
| 1. Hvor lang tid går der fra du vågner om morgenen, til du ryger eller bruger andre nikotinprodukter? *How much time passes from when you wake up in the morning until you smoke or use other nicotine products?* | - 0 – 15 min. - 15 min. 1- hour - More than 1 hour |
| 1. Hvornår ryger du, eller bruger andre nikotinprodukter  for sidste gang, inden du går i seng om aftenen? *When do you smoke or use other nicotine products for the last time before going to bed at night?* | - Yes - No |
| 1. Føler du, at du har svært ved at koncentrere dig, bliver rastløs, nervøs, nedtrykt eller irritabel, når du ikke har røget eller brugt andre nikotinprodukter i et stykke tid? *Do you feel it’s hard to ocncentrate, or du you become restless, down, or irritable when you haven’t smoked or had nicotine for a while?* | - Yes - No |
| 1. Får du nogensinde en sådan trang til at ryge eller bruge andre nikotinprodukter, at du har svært ved at tænke på andet? *Do you ever feel such a strong urge to smoke or use other nicotine products that it's hard to think about anything else?* | - Yes, always - Yes, sometimes - No |

To qualify the experience from the feasiblity municipalities the score was tested in mini-interviews with 5 young adults aged between 19 and 30. Based on the experiences from the feasibility municipalities and the second round of face validation no further changes were made.

A thorough evaluation of the score is planned but is not a part of this current study of the effectiveness of the overall intervention.

# References

[1] DiFranza JR. Initial symptoms of nicotine dependence in adolescents. *Tob Control* 2000; 9: 313–319.

[2] Prokhorov AV, Khalil GE, Foster DW, et al. Testing the nicotine dependence measure mFTQ for adolescent smokers: A multinational investigation. *Am J Addict* 2017; 26: 689–696.

[3] Taherdoost H. Validity and Reliability of the Research Instrument; How to Test the Validation of a Questionnaire/Survey in a Research. *SSRN Electron J*. Epub ahead of print 2016. DOI: 10.2139/ssrn.3205040.

[4] Aithal A, Aithal PS. Development and Validation of Survey Questionnaire & Experimental Data – A Systematical Review-based Statistical Approach. *Int J Manag Technol Soc Sci* 2020; 233–251.
