# Supplementary material for: Effectiveness of the U-Niko intervention: Protocol for a cluster randomized controlled trial of a municipal-based tobacco and nicotine cessation intervention for adolescents and young adults
Source: PLoS One. 2025 Oct 16;20(10):e0323514. doi: 10.1371/journal.pone.0323514 (PMC12530545; doi:10.1371/journal.pone.0323514)
Supplement: S5 — (PDF) [file pone.0323514.s005.pdf]

Tobacco and nicotine product cessation for young people (project U-Niko) – A nationwide cluster randomized trial

Charlotta Pisinger & Sofie SB Rasmussen

Introduction

In recent years, there has been an increase in adolescents' and young adults use of tobacco- and nicotine products. Almost 36% of the Danish adolescents and young adults between 15 and 29 years use at least one tobacco or nicotine product [1].

Smoking has a negative impact on both the physical and the mental health and school absence in youth [2–7]. Early exposure to nicotine might have long-term negative consequences on the brain[3–5,8–11] and also seems to be a so-called "gateway drug" to other psychoactive substances such as cocaine and cannabis [12–17]. Therefore, youths should be urged to stop using nicotine-containing products as early as possible.

Most Danish adolescents and young adults wish to quit

More than half of the young people who smoke wish to quit [1], and the proportion that has tried to quit in the last 12 months is higher among young people compared to older people who smoke [18]. Almost three out of four young users of oral nicotine products have considered quitting within the last month [1]. They should be incited to stop on their own, but unfortunately, many young users of tobacco and nicotine products are highly addicted [19]. The mean Fagerström nicotine dependence score measured among young people who attended smoking cessation counseling in the Danish municipalities was high (between 5.2 and 5.4) [20]. In 2021, approx. one out of four of the 16 to 25-year-old daily smokers reported that they wished to receive help to quit smoking.

Insufficient smoking cessation support for young people in Denmark

Denmark has developed cost-free, high-quality smoking cessation services targeted at all smokers. Group-based smoking cessation counseling is the most frequently used, and it is very effective [21–25]. Unfortunately, young people make very little use of these municipal evidence-based smoking cessation services and have lower quit rates than adults [26]. Only 407 young people between 15 and 24 years (i.e., less than 5‰ of the young smokers in that age group in Denmark) have received professional support for smoking cessation in 2023.

In a nationwide survey conducted by the research group in the pilot study (see Supplementary material, file 1), four in five municipalities reported having no/very low/low experience with youth-oriented quit services. Nine out of ten municipalities found it difficult to recruit youth to cessation services and to help them achieve abstinence [26,27]. The municipal counselors called for better education, new recruitment strategies, and new quit materials aimed at the often very addicted young people. Our findings suggest that the cessation services, which have been designed for and are very efficient for adult smokers, seem to have failed to meet the needs of young smokers and users of novel nicotine products, at least in Denmark [26].

Low evidence on tobacco and nicotine cessation interventions for adolescents and young adults

The published literature has been found to be *inconclusive,* but interventions based on s*ocial cognitive theory* seem to be effective in promoting longer-term abstinence in young adults[28]. Another review concluded that the quality of evidence was low or very low but that there is some evidence of the effect of *group-based smoking cessation counseling* for adolescents [29]. Pharmacotherapy seems to be well tolerated by young people, but no significant effect has been shown [30 31]. Regarding cessation of nicotine products (not cigarettes): Low-grade evidence suggests that *multi-component interventions that include counseling and peer elements* and a text-based cessation program*, based on behavioral*

support, may be effective in the cessation of nicotine products in adolescents and young adults [32]. For details, please see Supplementary material, file 2.

The U-Niko intervention

The intervention is centered around the needs of the youth and the municipalities who, in Denmark, have the responsibility of helping them quit. The intervention is developed based on state-of-the-art international research, results of a nationwide survey, interviews with smoking cessation counselors from the whole country, and observations and interviews with young users of tobacco- and nicotine products (Supplementary material, file 2).

The intervention consists of three areas:

A. A strategy to recruit adolescents and young adults to smoking and nicotine cessation services
B. Training of counselors who want to help young smokers and nicotine product users to quit
C. A tobacco- and nicotine cessation program for adolescents and young adults

Even if a very effective tobacco- and nicotine cessation intervention were developed, it would not have an impact on young people's tobacco and nicotine use if we did not have trained counselors who could deliver it and if we could not recruit young people. We hypothesize that there will be a synergistic effect by optimizing all three areas at the same time.

Objectives

Project U-Niko is the first-ever randomized controlled trial to evaluate the effectiveness of a tobacco- and nicotine cessation intervention for young people in a Danish municipal setting. The study aims to develop and test the effect of a tobacco- and nicotine cessation intervention for adolescents and young adults.

The primary aim of the study is to evaluate the effectiveness of this three-legged intervention aimed at adolescents and young adults compared with a control condition. The secondary aims are to examine the implementation level, adherence, and satisfaction with key factors in each intervention area as hypothesized mediators of intervention effectiveness. Furthermore, we want to examine the usefulness of a new score for assessing nicotine craving and abstinence symptoms (the U-Niko score) from all tobacco and nicotine products, including dual/poly use. The Danish counselors have expressed a need for this.

We hypothesize that youths in the intervention group will be more likely to be abstinent from all tobacco and nicotine products at the 6-month follow-up compared to youths in the control group.

Methods

The study we apply for is the third step of a planned nationwide intervention trial. The first step, the pilot study, and the second step, the feasibility study, have been completed, and we have followed the schedule as intended (Supplementary material, file 1).

The study is a cluster randomized trial that will take place in 55 Danish municipalities from January 1 to December 31, 2025. The municipalities have been randomized to a U-Niko intervention group (n=27) or a control group (n=28). The adolescent and young adult participants in the U-Niko project will receive either the intervention or the control condition based on the municipality in which they live, go to school, work, or in some other way have their daily activities.

The study will be conducted in accordance with the Danish Council for Independent Research's ethical guidelines. The study will be approved in accordance with the Data Protection Regulation and comply with the General Data Protection Regulation (GDPR) (EU) 2016/679. Parental permissions are not

required by the Ethical Committee for minors aged 15 or over since this study is behavioral-oriented and does not include a collection of biological materials.

Target group (inclusion criteria)

Adolescents and young adults aged 16 to 25 years who are currently using at least one tobacco- or nicotine product are interested in quitting and want assistance to quit. The young people will come from all socioeconomic groups and have different gender and ethnic backgrounds. We assume that at least 600 young people will get assistance to quit all over Denmark and that many more young people, as a "side-effect," will be nudged to try to quit on their own (not a part of the evaluation).

Exclusion criteria

Participants are excluded from the study if they fail to provide contact information and informed consent during the baseline assessment process.

Randomization

We invited all Danish municipalities to register for the trial. In total, 55 municipalities accepted the invitation and were enrolled and randomized into the intervention group or the control group using the online randomization program Sealed Envelope [33]. During the randomization, we stratified the municipalities according to municipal size and according to self-reported close collaboration between municipalities (health clusters). The latter was done to ensure that there would be no spill-over effect from the intervention groups to the control groups during the intervention year. The randomization was blinded.

The intervention municipalities

All young participants will, at baseline (first session), complete a survey on personal characteristics and information on tobacco- or nicotine use, as well as the U-Niko scores for craving and withdrawal symptoms. The score will be used to estimate the eventual need for nicotine replacement therapy. The score consists of 5 questions and ranges from 0-9, where 9 is the highest score for estimated abstinence. Further, all participants who are self-reported tobacco- and nicotine-free at six months follow-up will be invited to do a saliva cotinine test to validate their abstinence. See a detailed illustration of the intervention activities below (Figure 1).

Figure 1: Intervention flowchart

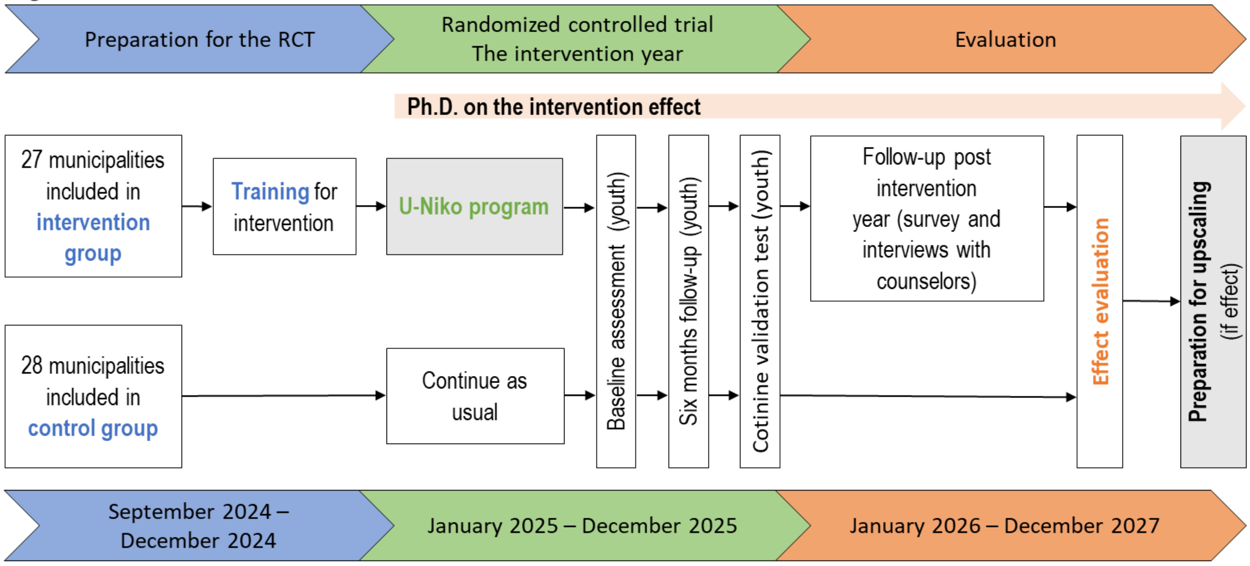

Training of youth counselors

Each municipality is encouraged to find two to three persons to receive the U-Niko youth counselor training program. The choice of counselors is important. They must be motivated to work with young people and have a feeling of good connection with young people. The new youth counselors might be existing smoking cessation counselors, but they can also be teachers, pedagogues, or people working with addiction. They do not need a health-related education, and we believe that it will be an asset for many schools and workplaces to educate their youth coaches, who are experienced in working with young people. The training is spread over three days and is planned to take place in the fall of 2024 so that the municipalities are trained and ready to recruit young participants for cessation courses at the very beginning of the intervention year 2025.

The recruitment strategy

The recruitment program is divided into four recruitment steps:

1. *Where do you recruit young people?*
   The U-Niko program offers step-by-step guidance on how to establish collaborations with youth arenas, i.e., schools, workplaces, fitness centers, sports clubs, and so on, where the young people are, as well as guidance on how to contact and establish collaborations with youth arenas. The program offers videos and letters to the head of the arena.
2. *How do you meet and recruit the young people?*
   The program offers guidance and examples of how to promote the course on tobacco- and nicotine cessation in a motivating way using face-to-face interactions in the arena and tools such as videos with peers reflecting on their use of tobacco/nicotine products and their wish to quit and posters with peers and photos of oral lesions (due to snus use).
3. *How do you ensure that the young people participate in the course after registration?*
   Keeping in touch with young people and strengthening their motivation is an important part of the recruitment process. Recommendations and examples on how to do this are given.
4. *How do you expand your good work?*
   The final part of the recruitment strategy focuses on how to strengthen existing collaboration with an arena, e.g., a high school, and how to expand to other arenas, e.g., boarding schools, sports clubs, or workplaces, year by year.

The tobacco- and nicotine cessation course

The course consists of seven group-based counseling sessions over eight weeks. The first three sessions are planned to be motivation building and preparation for the target quit date and potential high-risk situations. The four sessions planned after the quit date are focused on abstinence, how to deal with the symptoms, and keeping up the motivation to stay nicotine-free.

Approx. 12 young people are recruited to a group, and we expect that 8 of these will attend the course, which is the recommended size of participants.

The course has six focus areas:

- *Long-term abstinence from all tobacco and nicotine products*
  The participants must commit to a full cessation since there is no evidence that a reduction in use has a benefit on mortality or that it increases the probability of cessation in the longer term.
- *Behavioral counseling*
  International literature and experiences from our feasibility study found that behavioral counseling is effective in helping young people quit using tobacco and nicotine products.
- *A pedagogical and relation-building approach*
  The program focuses on building a good and trust-based relationship with the young people and on counseling the group so all participants feel safe and receive the attention they need.
- *Peer elements*
  International literature and experiences from our feasibility study suggest that including peer

elements in the cessation process can be effective [34–37]. Therefore, the course is group-based, and we include peer elements, such as videos of other young people's cessation processes.

- *Focus on addiction*
  The program concentrates on providing young people with an understanding of their addiction and tools and persistent motivation to overcome their addiction. The level of nicotine addiction and risk of craving and withdrawal symptoms will be tested using the newly developed U-Niko score. As there is no/very weak evidence of the effect of cessation pharmacotherapy for young people, only those with high scores on the scale are recommended to use a nicotine patch.
- *Text messages to encourage and remind the participants about cessation activities*
  There is experience and some evidence that shows that encouragement and frequent reminders by text messages can diminish drop-out and increase quit rates among young people in cessation counseling [38].

In the feasibility study, we tested online counseling sessions, but the young people strongly preferred face-to-face counseling.

The control municipalities

The control municipalities will continue "as usual" and will not be provided with any knowledge about the intervention or assistance to improve their youth-oriented cessation activities. However, the control municipalities will be instructed on how to complete the same baseline assessment as the intervention municipalities for all young participants in their tobacco- and nicotine cessation activities (Figure 1). The instructions will be given via an online meeting, as well as video guides. Further, we will carry out the same procedure for follow-up and cotinine validation as in the intervention municipalities.

Data collection and analysis

Baseline characteristics, including tobacco- and nicotine use, will be registered in the Danish National Smoking Cessation Database, named Stopbasen (previously named Rygestopbasen). Stopbasen has high validity, and (almost) all cessation activities in Denmark are registered in this database [39]. The counselors report routinely to the database, and there is a special form used for young people. The characteristics of the participants, the number of young people recruited, and the outcomes of recruitment and short- and long-term abstinence rates will be registered by both the intervention and the control municipalities. The six-month follow-up on tobacco- and nicotine abstinence rates will be conducted via short telephone interviews in collaboration with Stopbasen. Cotinine validation testing will be done via a video call. The youth participating in the validation will receive a cotinine saliva test in the mail and be invited to take the test on a video call with a research student assistant [40].

The effectiveness of the training of counselors and several of the other secondary outcomes, e.g., measuring the level of implementation, will only be registered in the intervention group. All intervention municipalities will receive an online follow-up survey after the intervention year, and selected municipalities will be extracted for a more in-depth interview to evaluate the implementation process.

There will be no financial incentives for the municipalities or the youths participating in the study. However, a 300 DKK (approx. 40 Euro) gift certificate will be offered to participants for the cotinine validation, regardless of their test outcome.

Outcomes

We have a primary outcome for each of the three focus areas:

- **The training of new youth counselors:** A score based on youth counselors' self-reported perceptions of whether the training had provided sufficient knowledge and skills to work with the U-Niko program, measured at the end of the intervention year.

- **The recruitment strategy:** The number of 16 to 24-year-old persons recruited by a municipality to the stop program during the intervention year 2025.
- **The tobacco- and nicotine cessation course:** Self-reported point prevalence abstinence (not using any tobacco or nicotine product in the last 14 days) six months after the targeted quit date.

Further, we have secondary outcomes for each of the three intervention elements. The secondary outcomes are planned to nuance the primary outcomes and to contribute with analyses on intervention mediators as well as process evaluation of the implementation in the intervention municipalities. For an extended list of all secondary outcomes, please see Supplementary material, file 3.

Data analysis plan

Analyses of the intervention's primary outcomes will be conducted using mixed-effects linear and logistic regression models. We will adjust for all relevant confounding variables, and data will be presented with corresponding 95% confidence intervals. In the main abstinence analyses, we will apply the intention-to-treat (ITT) approach, assuming all non-respondents are current smokers/nicotine users. Further, we will take missing values into account by conducting a sensitivity analysis using a worst-case and a best-case approach. A two-sided *p*-value of <0.05 will be considered statistically significant.

Based on national data from Stopbasen, we estimate that the intervention group will see a treatment benefit of 11 percent points compared to the control group (23% vs. 34%). Sample size calculations where each municipality is considered a cluster with an inter-correlation coefficient (ICC) of 0.005 shows that each municipality must include at least 11 adolescents or young adults in their tobacco- and nicotine cessation activities during the intervention year. Outcomes on factors for implementation fidelity and satisfaction among participants will be analyzed using simple descriptive statistics and qualitative network analysis. For a more detailed analysis plan, see Supplementary material, file 4.

Key uncertainties

This study is a field trial, and many things can go wrong, as the intervention takes place in a real-life setting. The researchers have no impact on either recruitment or the course on tobacco and nicotine cessation that the youth counselors offer. Further, we do not offer extra resources to the municipalities. This was contemplated, but then the intervention would no longer reflect the real life in the municipalities. Also, it is key that the youth counselors engage not because a project pays them but because they are dedicated, find the training sufficient, the stop program meaningful, and that they have good experiences. Otherwise, they will not continue the program. The principals in schools, the managers of fitness centers and sports clubs, and shops where young people work, etc., are also important actors, as their cooperation is necessary. If they do not allow the young people to join the program, the coaches will fail.

The Danish Health Authorities launched a call in the spring of 2024. Municipalities interested in improving their work with the cessation of tobacco and nicotine products in adolescents and young adults could apply for funding. 58 million DKr. (approx. 7.7 million Euro) have been allocated to this work, and we know that many municipalities have applied for funding. The funding is to be used between fall 2024 and 2027. This might have a negative impact on our study, as some of the control municipalities might have a much higher activity level (recruit more young people) than usual. However, the Danish Health Authorities support the U-Niko project, encourage participation, and expect synergy. The call might also have a positive effect, as there is a greater attention on the cessation of tobacco and nicotine products in young people, and there will be more resources in some of the intervention municipalities. In our analyses, we will adjust for municipalities that received the extra funding.

Dissemination of knowledge

The goal of dissemination activities is to increase awareness of the project's experiences, and the target group is both national and international researchers, practitioners, and decision-makers. We will make use of relevant channels from the partners involved and of wider networks. We have given presentations on the project's experiences at national conferences and an international conference (Supplementary material file 1).

We have planned four papers as a part of this study. The papers will be published in a high-impact journal such as Tobacco Control, BMJ Public Health, or Frontiers of Public Health:

- A research protocol on the Project U-Niko cluster randomized controlled trial (fall of 2024)
- A paper on the main results of the effect of the U-Niko CRCT (winter of 2026/2027)
- A paper on the level of implementation of the U-Niko program and its impact on the results (spring of 2027)
- A paper on the new U-Niko craving and abstinence score (summer 2027)

Upscaling

If the intervention proves to be effective, we will apply for funding to upscale and implement all three focus areas in an online free-to-use version. We have very good connections with the municipalities, National Association of Local Authorities, Danish Cancer Society, Danish Heart Foundation, Healthy Cities Network's tobacco group, Danish Health Authorities, and other central partners when it comes to dissemination.

The online version will contain free educational videos, textbooks, and materials for the recruitment and the stop program, as well as step-by-step guides on how to implement and use the U-Niko materials. This online version of U-Niko will ensure that all who wish to work with youth tobacco- and nicotine cessation have the same free access to evidence-based resources and materials, and it will not require many resources to maintain.

Literature

1. Lund L, Jarlstrup N, Bast LS. *§RØG - En Undersøgelse Af Tobak, Adfærd Og Regler*. Statens Institut for Folkesundhed, SDU; 2024. Accessed May 6, 2024. https://www.sdu.dk/da/sif/rapporter/2024/roeg_rapport_5_tobak_regler_adfaerd

2. Yuan M, Cross SJ, Loughlin SE, Leslie FM. N icotine and the adolescent brain: Nicotine and the adolescent brain. *J Physiol*. 2015;593(16):3397-3412. doi:10.1113/JP270492

3. Smith RF, McDonald CG, Bergstrom HC, Ehlinger DG, Brielmaier JM. Adolescent nicotine induces persisting changes in development of neural connectivity. *Neurosci Biobehav Rev*. 2015;55:432-443. doi:10.1016/j.neubiorev.2015.05.019

4. Boden JM, Fergusson DM, Horwood LJ. Cigarette smoking and depression: tests of causal linkages using a longitudinal birth cohort. *Br J Psychiatry*. 2010;196(6):440-446. doi:10.1192/bjp.bp.109.065912

5. Moylan S, Jacka FN, Pasco JA, Berk M. Cigarette smoking, nicotine dependence and anxiety disorders: a systematic review of population-based, epidemiological studies. *BMC Med*. 2012;10(1):123. doi:10.1186/1741-7015-10-123

6. Chaarani B, Kan KJ, Mackey S, et al. Low Smoking Exposure, the Adolescent Brain, and the Modulating Role of CHRNA5 Polymorphisms. *Biol Psychiatry Cogn Neurosci Neuroimaging*. 2019;4(7):672-679. doi:10.1016/j.bpsc.2019.02.006

7. Perelman J, Leão T, Kunst AE. Smoking and school absenteeism among 15- to 16-year-old adolescents: a cross-section analysis on 36 European countries. *Eur J Public Health*. 2019;29(4):778-784. doi:10.1093/eurpub/ckz110

8. *The Health Consequences of Smoking - 50 Years of Progress: A Report of the Surgeon General*. National Center for Chronic Disease Prevention and Health Promotion (US) Office on Smoking and Health; :2014.

9. Vestbo J, Andreasen J, Bast L, Pisinger C. *Børn Og Unges Nikotinbrug - Konsekvenser Og Forebyggelse*. Vidensråd for Forebyggelse; 2022:1-96.

10. *E-Cigarette Use Among Youth and Young Adults. A Report of the Surgeon General*. U.S. Department of Health and Human Services; 2016.

11. Leslie FM. Unique, long-term effects of nicotine on adolescent brain. *Pharmacol Biochem Behav*. 2020;197:173010. doi:10.1016/j.pbb.2020.173010

12. Ren M, Lotfipour S. Nicotine Gateway Effects on Adolescent Substance Use. *West J Emerg Med*. 2019;20(5):696-709. doi:10.5811/westjem.2019.7.41661

13. Alajaji M, Lazenka M f., Kota D, et al. Early adolescent nicotine exposure affects later-life cocaine reward in mice. *Neuropharmacology*. 2016;105:308-317. doi:10.1016/j.neuropharm.2016.01.032

14. Huang YY, Kandel DB, Kandel ER, Levine A. Nicotine primes the effect of cocaine on the induction of LTP in the amygdala. *Neuropharmacology*. 2013;74:126-134. doi:10.1016/j.neuropharm.2013.03.031

15. Schwartz LP, Kearns DN, Silberberg A. The effect of nicotine pre-exposure on demand for cocaine and sucrose in male rats. *Behav Pharmacol*. 2018;29(4):316-326. doi:10.1097/FBP.0000000000000357

16. Linker KE, Gad M, Tawadrous P, et al. Microglial activation increases cocaine self-administration following adolescent nicotine exposure. *Nat Commun*. 2020;11(1):306. doi:10.1038/s41467-019-14173-3

17. McQuown SC, Belluzzi JD, Leslie FM. Low dose nicotine treatment during early adolescence increases subsequent cocaine reward. *Neurotoxicol Teratol*. 2007;29(1):66-73. doi:10.1016/j.ntt.2006.10.012

18. *Danskernes Rygevaner. Delrapport 3: Rygestop Og Rygestopadfærd*. Sundhedsstyrelsen; 2020.

19. Zettergren A, Sompa S, Palmberg L, et al. Assessing tobacco use in Swedish young adults from self-report and urinary cotinine: a validation study using the BAMSE birth cohort. *BMJ Open*. 2023;13(7):e072582. doi:10.1136/bmjopen-2023-072582

20. Tønnesen H, Rasmussen M. *STOPbasens temarapport: Unge i stopforløb - rygning, snus og e-cigaretter*. Zenodo; 2022. doi:10.5281/ZENODO.7600359

21. Neumann T, Rasmussen M, Heitmann B, Tønnesen H. Gold Standard Program for Heavy Smokers in a Real-Life Setting. *Int J Environ Res Public Health*. 2013;10(9):4186-4199. doi:10.3390/ijerph10094186

22. Neumann T, Rasmussen M, Ghith N, Heitmann BL, Tønnesen H. The Gold Standard Programme: smoking cessation interventions for disadvantaged smokers are effective in a real-life setting. *Tob Control*. 2013;22(6):e9-e9. doi:10.1136/tobaccocontrol-2011-050194

23. Kehlet M, Schroeder T, Tønnesen H. The Gold Standard Program for Smoking Cessation is Effective for Participants Over 60 Years of Age. *Int J Environ Res Public Health*. 2015;12(3):2574-2587. doi:10.3390/ijerph120302574

24. Rasmussen M, Klinge M, Krogh J, Nordentoft M, Tønnesen H. Effectiveness of the Gold Standard Programme (GSP) for smoking cessation on smokers with and without a severe mental disorder: a Danish cohort study. *BMJ Open*. 2018;8(6):e021114. doi:10.1136/bmjopen-2017-021114

25. Rasmussen M, Heitmann B, Tønnesen H. Effectiveness of the Gold Standard Programmes (GSP) for Smoking Cessation in Pregnant and Non-Pregnant Women. *Int J Environ Res Public Health*. 2013;10(8):3653-3666. doi:10.3390/ijerph10083653

26. Rasmussen SKB, Pisinger C. Nationwide experiences with youth-targeted smoking andnicotine product cessation. *Tob Prev Cessat*. 2023;9(August):1-13. doi:10.18332/tpc/169498

27. Rasmussen SB, Pisinger C, Koch MBB. *Kommunale Erfaringer Med Ryge- Og Nikotinstop Rådgivning Målrettet Unge*. Region Hovedstaden https://www.regionh.dk/forebyggelseslaboratoriet/publikationer/Documents/Kommunale%20erfaringer%20med%20ryge-%20og%20nikotinstopr%C3%A5dgning%20m%C3%A5lrettet%20unge_web.pdf

28. Park E, Zhou Y, Chen C, Chacko T, Mahoney M, Chang YP. Systematic review: interventions to quit tobacco products for young adults. *BMC Public Health*. 2023;23(1):1233. doi:10.1186/s12889-023-15900-8

29. Fanshawe TR, Halliwell W, Lindson N, Aveyard P, Livingstone-Banks J, Hartmann-Boyce J. Tobacco cessation interventions for young people. Cochrane Tobacco Addiction Group, ed. *Cochrane Database Syst Rev*. 2017;2017(11). doi:10.1002/14651858.CD003289.pub6

30. Myung SK, Park JY. Efficacy of Pharmacotherapy for Smoking Cessation in Adolescent Smokers: A Meta-analysis of Randomized Controlled Trials. *Nicotine Tob Res*. 2019;21(11):1473-1479. doi:10.1093/ntr/nty180

31. Gray KM, Rubinstein ML, Prochaska JJ, et al. High-dose and low-dose varenicline for smoking cessation in adolescents: a randomised, placebo-controlled trial. *Lancet Child Adolesc Health*. 2020;4(11):837-845. doi:10.1016/S2352-4642(20)30243-1

32. Rasmussen SB, Pisinger C. Nicotine product cessation interventions in young adults: a systematic review (Revised version submitted: 03/06/2024). *Tob Use Insights*.

33. Sealed Envelope: Randomisation and online databases for clinical trials. https://sealedenvelope.com/

34. Stotts RC, Roberson PK, Hanna EY, Jones SK, Smith CK. A randomised clinical trial of nicotine patches for treatment of spit tobacco addiction among adolescents. *Tob Control*. 2003;12 Suppl 4(Suppl 4):IV11-15. doi:10.1136/tc.12.suppl_4.iv11

35. Walsh MM, Hilton JF, Masouredis CM, Gee L, Chesney MA, Ernster VL. Smokeless tobacco cessation intervention for college athletes: results after 1 year. *Am J Public Health*. 1999;89(2):228-234. doi:10.2105/AJPH.89.2.228

36. Walsh MM, Hilton JF, Ellison JA, et al. Spit (Smokeless) Tobacco Intervention for High School Athletes. *Addict Behav*. 2003;28(6):1095-1113. doi:10.1016/S0306-4603(02)00228-9

37. Walsh MM, Langer TJ, Kavanagh N, et al. Smokeless tobacco cessation cluster randomized trial with rural high school males: intervention interaction with baseline smoking. *Nicotine Tob Res Off J Soc Res Nicotine Tob*. 2010;12(6):543-550. doi:10.1093/ntr/ntq022

38. Graham AL, Amato MS, Cha S, Jacobs MA, Bottcher MM, Papandonatos GD. Effectiveness of a Vaping Cessation Text Message Program Among Young Adult e-Cigarette Users: a Randomized Clinical Trial. *JAMA Intern Med*. 2021;181(7):923-930. doi:10.1001/jamainternmed.2021.1793

39. STOPbasen - tobak og nikotin. Accessed June 25, 2024. https://www.stopbasen.dk/

40. Salivary Cotinine: Quick Start Research Guide. Accessed June 25, 2024. https://salimetrics.com/analyte/salivary-cotinine/
